# TransRate: reference-free quality assessment of de novo transcriptome assemblies

**DOI:** 10.1101/gr.196469.115

**Published:** 2016-08

**Authors:** Richard Smith-Unna, Chris Boursnell, Rob Patro, Julian M. Hibberd, Steven Kelly

**Affiliations:** 1 Department of Plant Sciences, University of Cambridge, Cambridge CB2 3EA, United Kingdom;; 2 Department of Computer Science, Stony Brook University, Stony Brook, New York 11794-4400, USA;; 3 Department of Plant Sciences, University of Oxford, Oxford OX1 3RB, United Kingdom

## Abstract

TransRate is a tool for reference-free quality assessment of de novo transcriptome assemblies. Using only the sequenced reads and the assembly as input, we show that multiple common artifacts of de novo transcriptome assembly can be readily detected. These include chimeras, structural errors, incomplete assembly, and base errors. TransRate evaluates these errors to produce a diagnostic quality score for each contig, and these contig scores are integrated to evaluate whole assemblies. Thus, TransRate can be used for de novo assembly filtering and optimization as well as comparison of assemblies generated using different methods from the same input reads. Applying the method to a data set of 155 published de novo transcriptome assemblies, we deconstruct the contribution that assembly method, read length, read quantity, and read quality make to the accuracy of de novo transcriptome assemblies and reveal that variance in the quality of the input data explains 43% of the variance in the quality of published de novo transcriptome assemblies. Because TransRate is reference-free, it is suitable for assessment of assemblies of all types of RNA, including assemblies of long noncoding RNA, rRNA, mRNA, and mixed RNA samples.

High-throughput sequencing of RNA has revolutionized our ability to assess the genetic and quantitative basis of many complex biological traits. For organisms that have sequenced and annotated genomes, short reads can be directly mapped to these resources and quantitative estimates of gene expression (as well as splice-variants and mutations) can be determined using a variety of different methods. In the absence of an appropriate reference genome, de novo transcriptome assembly must be performed. These assemblies provide the primary data for gene discovery and evolutionary analyses, and facilitate quantitative assessment of differential gene expression. Given the importance of these applications to comparative biological research, several algorithms have been developed to produce de novo transcriptome assemblies from raw sequence data. Popular among these algorithms are Trinity (Grabherr et al. 2011), Oases (Schulz et al. 2012), Trans-ABySS (Robertson et al. 2010), IDBA-tran (Peng et al. 2013), and SOAPdenovo-Trans (Xie et al. 2014), each of which takes a different approach to the problem of reconstituting a transcriptome from short sequence reads. Furthermore, they all provide considerable flexibility with multiple parameters and heuristics that can be modified to allow the user to tailor assembly settings for variations in RNA-seq library construction, coverage depth, and differences between organisms. These large parameter spaces mean that the same read data can generate substantially different assemblies both within and between assembly methods. Likewise, altering parameter combinations can result in the assembly of contigs with varying properties such that disparate conclusions relating to gene content and expression level can be reached from the same input data.

In addition to the considerable algorithmic flexibility, the data being assembled can be generated from multiple different RNA types. These can range from specifically amplified subpopulations of particular types of RNA, to total RNA encompassing all RNA types within the cell. Given the wide range of input data and assembly methods, there is a need to be able to evaluate the quality of any de novo transcriptome in the absence of a known reference and identify the set of parameters or assembly methods that best reconstruct the transcriptome from which the raw read data was generated. Moreover, there is a need to be able to identify within a given assembly the set of contigs that are well-assembled from those that are not, so that incorrect data do not influence downstream biological interpretation.

Algorithms to assess the outputs of DNA-directed (e.g., genome and metagenome) assembly have been developed. These range in complexity from descriptive metrics (Gurevich et al. 2013) to explicit modeling of the sequencing and assembly process to provide a likelihood-based measure of assembly quality (Clark et al. 2013; Rahman and Pachter 2013). However, the assumptions used for evaluation of DNA-directed assembly such as uniformity of coverage (except in repetitive regions) and assembled contig length are not appropriate for the assembly of transcriptomes due to the exponentially distributed coverage of different transcripts and log-normally distributed transcript lengths. Therefore alternative criteria that are tailored for the biological properties of transcriptomes need to be used for the assessment of de novo assembled transcriptomes.

To date, the majority of de novo transcriptome assessment methods have exploited comparative approaches in which the assembled transcriptome is compared to a known reference data set (O'Neil and Emrich 2013; Lowe et al. 2014). These comparative methods provide insight into the complement of known proteins that are represented within a de novo assembly but do not reveal the extent to which the contigs representing those proteins are assembled correctly. Furthermore, due to the inherent limitations of such comparative analyses, they only assess the de novo transcriptome on the subset of contigs that represent conserved proteins. Highly divergent transcripts, novel transcripts, and noncoding transcripts are not assessed by these methods, and thus the assessment measures do not consider all the data. Moreover, de novo assembly of noncoding RNA or specific subpopulations of RNAs are poorly evaluated by these comparative methods. To date, only a single reference-free transcriptome assembly evaluation tool has been produced, RSEM-eval (Li et al. 2014). RSEM-eval provides an assembly likelihood given the read data, allowing the comparison of assemblies generated from the same input data. Although RSEM-eval quantifies the relative contribution that each contig makes to an overall assembly score, it does not provide descriptive statistics about the quality of contigs within an assembly.

Here, we present TransRate, a novel method for evaluation of the accuracy and completeness of de novo transcriptome assemblies. TransRate assesses these features through two novel reference-free statistics: the TransRate contig score and the TransRate assembly score. The TransRate contig score provides a quantitative measure of the accuracy of assembly for each individual contig, and the TransRate assembly score provides a quantitative measure of the accuracy and completeness of the assembly.

## Results

### Problem definition and approach

**Figure 1.**
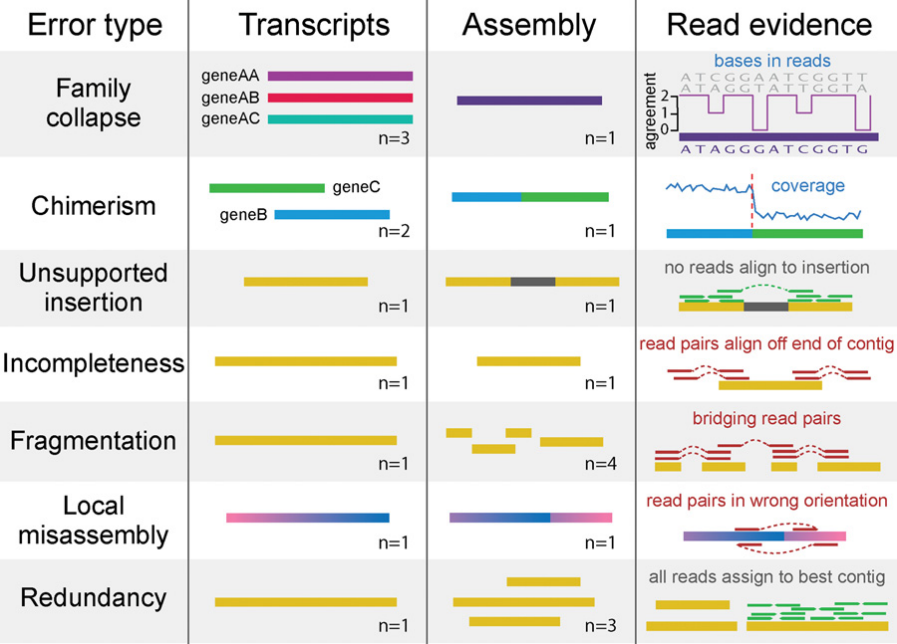
Common errors in de novo transcriptome assembly, and how they can be detected using read mapping data. Family collapse occurs when multiple members of a gene family are assembled into a single hybrid contig. This error can be detected by measuring the extent that the nucleotides in the contig are supported by the mapped reads. Chimerism occurs when two or more transcripts (that may or may not be related) are concatenated together in a single contig during assembly. This can be detected when the expression levels of the transcripts differ, leading to a change-point in the read coverage along the contig. Unsupported insertions can be detected as bases in a contig that are unsupported by the read evidence. Incompleteness can be detected when reads or fragments align off the end of the contig. Fragmentation is caused by low coverage and is detectable when read pairs bridge two contigs. Local misassembly encompasses various structural errors that can occur during assembly, such as inversions, usually as a result of assembler heuristics. These are detectable when both members of a read pair align to a single contig, but in a manner inconsistent with the sequencing protocol. Redundancy occurs when a single transcript is represented by multiple overlapping contigs in an assembly. This is detectable when reads align to multiple contigs but the assignment process assigns them all to the contig that best represents the original transcript.

The aim of de novo transcriptome assembly is to accurately reconstruct the complete set of transcripts that are represented in the read data in the absence of a reference genome. There are several contributing factors that negatively affect the accuracy of this reconstruction process. These factors include error in the sequencing process, incomplete coverage of transcripts (due to insufficient sequencing depth), and real biological variability (such as variation in exon/intron retention, variation in exon boundary usage, and variation in nucleotide sequence between alleles). Moreover, assembly errors can originate from algorithmic simplifications (such as representing the information contained in the reads as shorter words) and allowances (e.g., permitting assembly of fragments containing mismatches) that are used to mitigate the computational complexity of the assembly problem. Together, these factors cause several common assembly artifacts, including hybrid assembly of gene families, transcript fusion (chimerism), spurious insertions in contigs, and structural abnormalities such as incompleteness, fragmentation, and local misassembly of contigs (Fig. 1).

**Figure 2.**
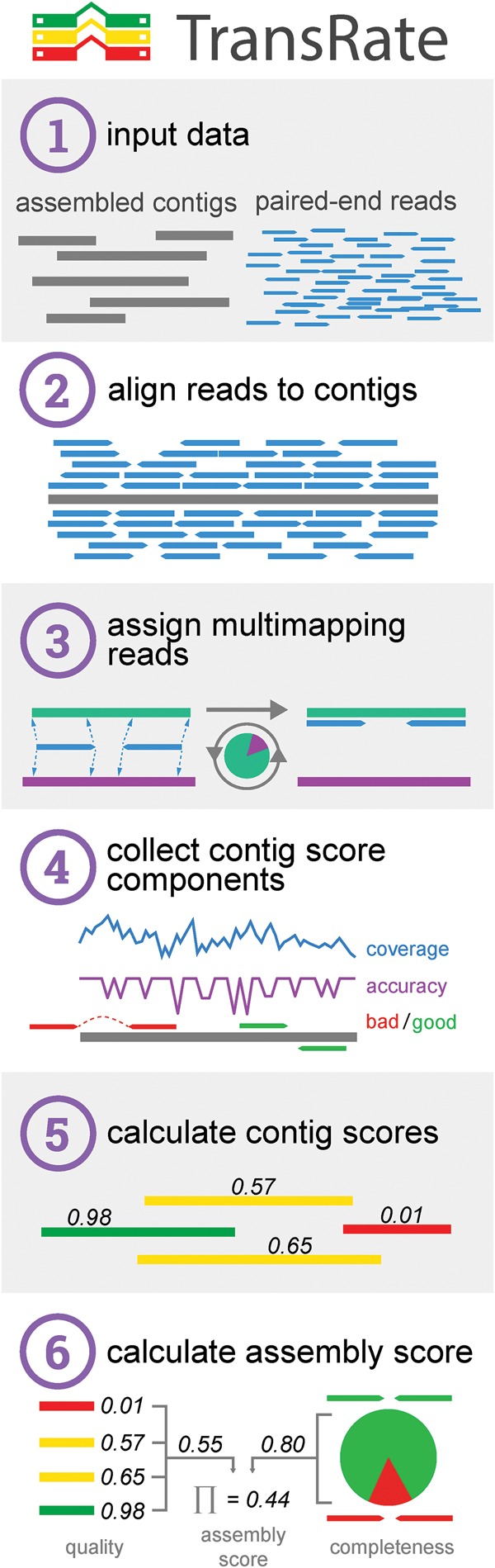
The TransRate workflow. (1) TransRate takes as input one or more de novo transcriptome assemblies and the paired-end reads used to generate them. (2) The reads are aligned to the contigs. (3) Multimapping reads are proportionally assigned to contigs based on the posterior probability that each contig was the true origin of the read. (4) The alignments are evaluated using four discrete score components. (5) The four score components are integrated to generate the TransRate contig score. (6) The TransRate assembly score is calculated from analysis of all contig scores.

TransRate is focused on a clear problem definition, i.e., to assess the accuracy and completeness of a de novo assembled transcriptome using only the input reads. TransRate proceeds by mapping the reads to the assembled contigs, proportionally assigning multimapping reads in a probabilistic manner to their contig of origin, analyzing the alignments, calculating contig-level metrics (Table 1), integrating these contig-level metrics to provide a contig score, and then combining the completeness of the assembly with the score of each contig to produce an overall assembly score (Fig. 2). TransRate also provides an abundance weighted assembly score which weights each constituent contig score by the relative abundance level of each contig.

**Table 1.**
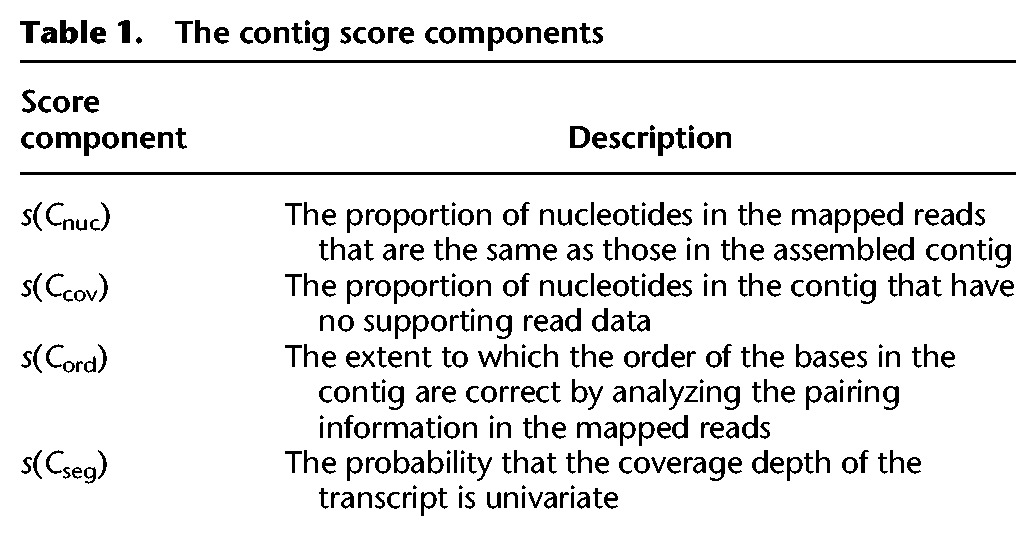
The contig score components

### Contig assessment criteria

To calculate the TransRate contig score, a correctly assembled contig is assumed to have the following four properties: (1) The identity of the nucleotides in the contig will accurately represent the nucleotides of the true transcript; (2) the number of nucleotides in the contig (i.e., the assembled transcript length) will accurately represent the number in the true transcript; (3) the order of the nucleotides in the contig will accurately represent the order in the true transcript; and (4) the contig will represent a single transcript. We propose that each of these four statements can be approximated through analysis of the reads that map to the assembled contigs and are encapsulated by the four metrics presented in Table 1. For a detailed description of these metrics and how they are calculated, see the “TransRate contig scores” section in Methods.

**Figure 3.**
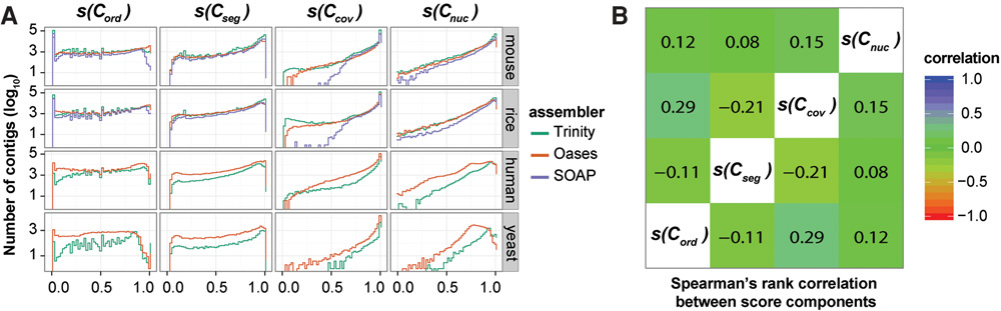
Distribution and interrelationship of contig score components. (*A*) Distribution of contig score components in 10 different assemblies spanning four species and three different assemblers. *s*(*C*_nuc_) is the fraction of nucleotides in a contig whose sequence identity agrees with the aligned reads. *s*(*C*_cov_) is the fraction of nucleotides in a contig that have one or more mapped reads. *s*(*C*_ord_) is the fraction of reads that map to the contig in the correct orientation. *s*(*C*_seg_) is the probability that the read coverage along the length of the contig is best explained by a single Dirichlet distribution, as opposed to two or more distributions. (*B*) The Spearman's rank correlation coefficient between the contig score components, averaged across all species and assemblers.

To determine whether these four contig-level metrics were discrete, and thus captured different properties of each assembled contig, their performance was evaluated on a range of assemblies generated using different algorithms from multiple different species (Fig. 3A). For each contig-level metric, the distributions of observed scores was broadly similar irrespective of species or assembly algorithm (Fig. 3A). One notable exception to this observation is that the distribution of *s*(*C*_cov_) (Table 1) observed for rice and mouse contigs generated using SOAPdenovo-Trans was markedly different to that observed for Oases and Trinity for the same species. This reveals that the contigs generated using SOAPdenovo-Trans on this rice data contained fewer regions that had zero coverage after read mapping.

Visual inspection of the global behavior of the contig-level metrics suggested that the four scores could be classified into two groups based on the density function of the observed score values. Both *s*(*C*_ord_) and *s*(*C*_seg_) (Table 1) produced approximately uniform distributions spanning the entire score range (Fig. 3A), whereas *s*(*C*_cov_) and *s*(*C*_nuc_) (Table 1) produced distributions whose density increased toward higher values (Fig. 3A). To determine whether these visually similar distributions were correlated, and thus measured features of the assembled contigs that were interdependent, we analyzed the pairwise Spearman's rank correlation between the score components. This revealed that the metrics were poorly correlated (Fig. 3B), and thus each provided discrete assessment of the assembled contigs to which they were applied.

Manual inspection of reference-based results for the 30 lowest-scoring contigs according to each score component was consistent with the individual score components capturing their target properties (Supplemental Fig. S1). The Bayesian segmentation of coverage depth, *s*(*C*_seg_), was also evaluated by inspection of coverage depth profiles (Supplemental Fig. S2) and simulation of artificial transcript chimeras. The latter was done by in silico fusion of randomly selected transcripts from the yeast transcriptome and assessment of *s*(*C*_seg_) scores as a function of the difference in abundance between the fused transcripts (Supplemental Fig. S3). Here, the segmentation method was unable to distinguish chimeras between transcripts whose abundance differed by less than twofold (Supplemental Fig. S3). The individual score components are provided in the TransRate program output so that end users can gain insight into the common sources of error in their assembly.

### Evaluation of the TransRate contig score

**Figure 4.**
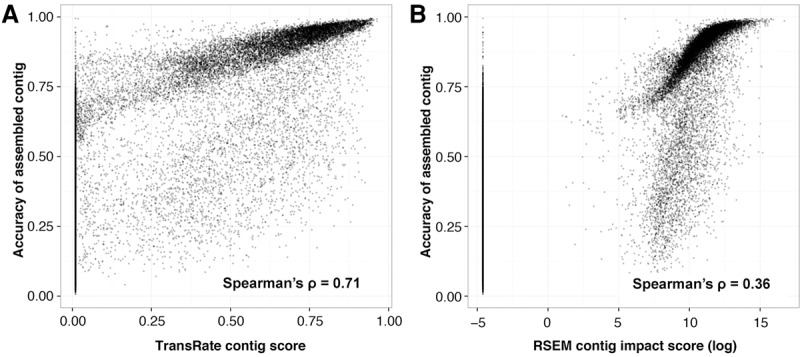
TransRate contig score is related to assembly accuracy. Contigs from assemblies of simulated reads from four species (rice, mouse, yeast, and human) were evaluated using TransRate and RSEM-eval. Reciprocal best-BLAST against the true set of transcripts was used to determine the *F*-score, or reference-based accuracy, of the assembled contig. Each point is a contig in an assembly, with all four assemblies on the same plot. (*A*) Comparison between TransRate contig score and contig *F*-score. (*B*) Comparison between RSEM-eval contig impact score and contig *F*-score, with contig impact scores below 0 set to the smallest positive value in the data to enable plotting.

As the contig-level metrics provided discrete evaluation of assembled contigs, we sought to determine whether the geometric mean of these metrics (see Methods, equation 1) was informative of the accuracy of assembly. To assess this, 4 million read pairs were simulated from each of the four test species (rice, mouse, human, and yeast; see Methods, “Independence of score components”) and assembled using SOAPdenovo-Trans with default settings. Simulated reads were used here so that the true set of transcripts was known and hence the accuracy of the assembled contigs could be assessed. The resultant assemblies were subjected to TransRate assessment, and the utility of the TransRate contig scores was assessed by comparing them to a conventional measure of contig accuracy calculated by alignment of the assembled contigs to the transcripts used to simulate the reads (see Methods, “Calculation of contig accuracy”). Comparison of these measures revealed that there was a strong monotonic relationship between contig accuracy and TransRate contig score (Fig. 4A). Across all simulated data sets, the TransRate contig score exhibited a Spearman's rank correlation with contig accuracy of ρ = 0.71 (Fig. 4A; Supplemental Table S1). For comparison, we also applied RSEM-eval to the same data set (Fig. 4B). Here, the contig impact score from RSEM-eval, which measures the relative contribution of every contig to the assembly score, also showed a positive correlation with contig accuracy; however, the Spearman's rank correlation with accuracy was lower than that observed for TransRate (ρ = 0.36) (Supplemental Table S1). Nonparametric correlation measures were used here to enable unbiased comparison of TransRate and RSEM-eval scores because their score distributions differ in type, location, scale and shape.

Analysis of the interrelationship between contig scores and contig accuracy revealed that both assessment methods exhibited minimum value inflation (Fig. 4A,B). Although some of these minimum value contigs comprise accurately assembled transcript sequences, they are assigned minimum score values as they fail to acquire mapped reads during the read-mapping process. This occurs due to the presence of contigs within the assembly that better represent the true contig than the contig in question and thus preferentially obtain all of the mapped reads during the probabilistic read assignment stage. This phenomenon commonly occurs when the contig in question is a substring of a longer contig in the assembly. As these contigs are redundant and they would be quantified as “not expressed” in downstream expression analyses of the assemblies, both TransRate and RSEM-eval are justified in the assignment of minimum value scores to these contigs. In the absence of these minimum value contigs, the Spearman's correlation coefficients for both TransRate and RSEM-eval are ρ = 0.70 and ρ = 0.77, respectively.

### Application of TransRate for relative evaluation of de novo assemblies from the same read data

**Figure 5.**
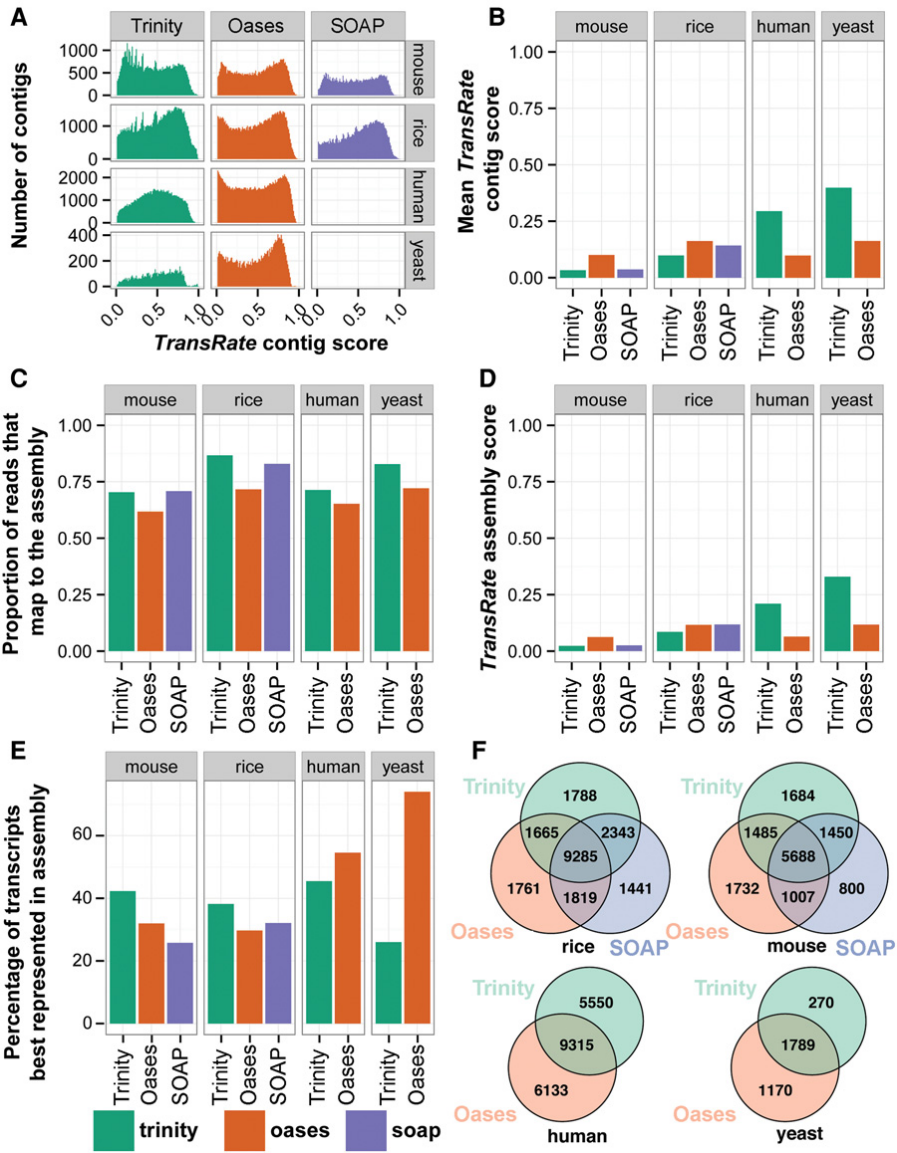
Calculation of TransRate assembly scores. (*A*) Distribution of TransRate contig scores for the 10 representative assemblies from real data. (*B*) Geometric mean of TransRate contig scores for all assemblies. (*C*) Proportion of reads that map to each assembly. (*D*) Final TransRate assembly scores for the 10 representative assemblies. (*E*) The proportion of reference transcripts that are best assembled by individual assembly methods. (*F*) The number of reference transcripts (identified by reciprocal best BLAST) that are assembled by each assembler.

Because the TransRate contig score is strongly related to contig accuracy, we sought to develop an assembly-level score that summarized the information captured by assessment of the individual contigs (Fig. 4A). Here, the geometric mean of all contig scores was selected such that each contig contributed equally to the final assembly assessment (see Methods, equation 2). Analysis of the TransRate contig score distributions for assemblies generated using different assembly algorithms from different species revealed that most assemblers produced contigs that obtained a wide range of scores (Fig. 5A). Some distributions also appeared to be multimodal with overlapping populations of low and high scoring contigs (Fig. 5A).

Comparison of the geometric mean of the contig scores revealed that on different data sets, different assemblers tended to produce more accurate assemblies (Fig. 5B). On average, Oases (version 0.2.06 with Velvet version 1.2.07) produced the highest mean contig scores for mouse and rice, whereas Trinity (version Trinity-r2013-02-25) produced the highest mean contig scores for human and yeast (Fig. 5B). The percentage of the input that could be mapped to these assemblies ranged from 65% to 85%, and thus, significant amounts of read data failed to be assembled by each method (Fig. 5C). To provide a single assembly assessment score that combined the proportion of read data contained within the assembly and the mean accuracy of the constituent contigs, we took the product of the geometric mean contig score and the proportion of reads mapping to the assembly (Fig. 5D). This assembly score places equal importance on the accuracy of each of the assembled contigs and the proportion of the input read data that is captured by the de novo assembly. In an ideal scenario, in which all of the input reads map back to the assembled contigs with no disagreement between the reads and the assembly, the assembly score will be 1. Errors in the sequencing or assembly process that cause reads to be omitted from the assembly or reads to disagree with the assembled contigs will cause the assembly score to tend toward 0.

TransRate also provides an abundance-weighted contig score (see Methods, equation 3), in which transcripts with assembly errors are penalized in proportion to their abundance. That is, highly abundant transcripts with errors are penalized more heavily than low abundance transcripts with the same errors. Using these abundance-weighted contig scores, an abundance-weighted assembly score can also be evaluated (see Methods, equation 4). The results from using these abundance-weighted scores exhibit the same trend as for the TransRate contig and assembly scores (Supplemental Fig. S4). However, the additional penalty due to abundance weighting causes the overall scores to be much lower (Supplemental Fig. S4). Caution should be exercised by the user when using the abundance-weighted contig scores because they are not comparable between contigs. That is, a highly abundant transcript with an assembly error will have a lower score than a transcript with the same error that is expressed to a lower level.

### Further comparison of de novo assemblies using BLAST and TransRate

To demonstrate additional ways in which TransRate can be combined with BLAST-based assessment of de novo transcriptome assemblies, the de novo assemblies were annotated using reciprocal best BLAST (bidirectional best BLAST hit) against the appropriate Ensembl reference data set for each species. The TransRate scores for these contigs were compared, and the proportion of transcripts that had the highest TransRate score for each assembly was recorded (Fig. 5E). No one method consistently outperformed the others; rather, the different assemblers produced the best assembly for >25% of transcripts (Fig. 5E). Analysis of the total number of reference transcripts that were assembled by the different methods revealed that, although there was significant agreement between the methods, each method uniquely assembled a large number of bona fide transcripts not assembled by the other methods (Fig. 5F). Taken together, these analyses lend support to the idea that combining contigs from multiple assembly methods is an effective way to increase the completeness of a de novo assembled transcriptome.

### Filtration of contigs using TransRate contig scores

Figures 4, A and B, and 5A show many contigs within a given assembly can achieve low or minimum value scores, and thus users may desire to remove them from the assembly. Although TransRate allows the user to specify any contig score cut-off between 0 and 1 for filtration of assembled contigs, it also provides an alternative option whereby a specific contig score cut-off can be learned for any given assembly. To do this, TransRate uses a global optimization method to find the contig score cut-off value such that the TransRate assembly score function is maximized (Supplemental Fig. S5). This automated cut-off method is consistent with the problem definition and overall aim of TransRate (to assess the accuracy and completeness of a de novo assembled transcriptome using only the input reads) as it automatically selects the subset of contigs that maximizes both accuracy and completeness. It should be noted that filtering contigs in this way may remove some accurately assembled low abundance transcripts that have incomplete coverage.

To provide an example of the results obtained from the application of the automated TransRate contig filtering, the 10 assemblies analyzed in Figure 5 were subject to filtering. Those de novo assembled contigs that contained regions with >95% identity to predicted genes in the genomes of the source species were selected for further analysis. On average, 20% of genes that had contigs matching at least part of a predicted gene were filtered out by TransRate (Supplemental Fig. S6). Of the genes whose entire length was encompassed in a single transcript, ∼12% were discarded by TransRate (Supplemental Fig. S6). Although TransRate has identified these transcripts as poorly assembled, and caution should be exercised against using abundance level estimates for these contigs, they may contain regions that have utility in certain analyses (e.g., phylogenetic analysis).

### Comparative analysis of 155 published assemblies provides a reference for calibration and elative assessment of assembly quality

**Figure 6.**
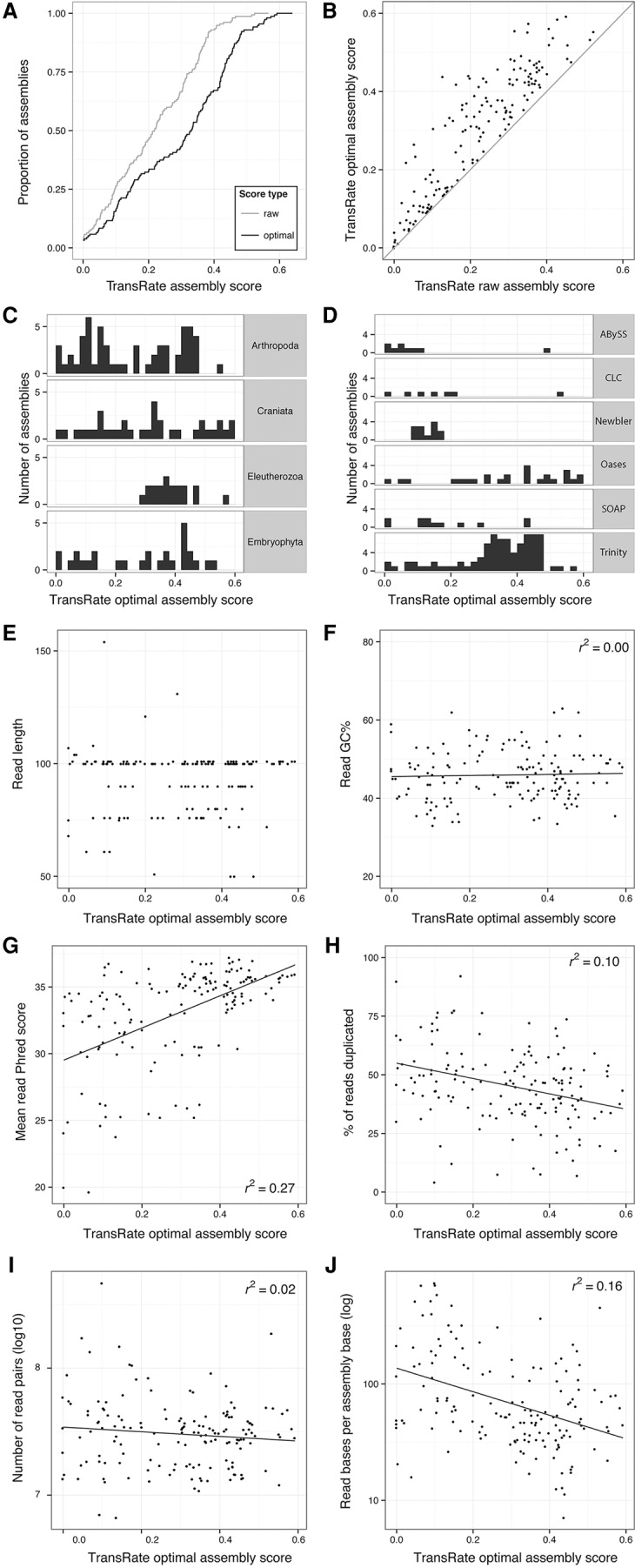
Application of TransRate to 155 published assemblies from the NCBI Transcriptome Shotgun Archive. One hundred fifty-five assemblies from the Transcriptome Shotgun Archive were analyzed using TransRate. The quality of the reads used to generate the assemblies were also analyzed using FastQC. (*A*) Cumulative distribution of TransRate raw and optimized assembly scores for each of the 155 assemblies. (*B*) Comparison between raw and optimized assembly score. (*C*) Distribution of TransRate optimized assembly scores partitioned by taxonomic group. (*D*) Distribution of TransRate optimized assembly scores partitioned by assembly method. (*E*–*J*) TransRate optimized assembly scores compared to various summary statistics of the input reads: (*E*) read length; (*F*) read GC%; (*G*) mean read per-base Phred score; (*H*) percent of reads that were PCR duplicates; (*I*) number of read pairs; and (*J*) read bases per assembled base.

To provide a reference distribution of TransRate assembly scores that end users can use to assess the relative merit of their own assemblies, TransRate was applied to a set of 155 published de novo assembled transcriptomes (Supplemental Table S2). All assembled transcriptomes were downloaded from the NCBI Transcriptome Shotgun Archive (http://www.ncbi.nlm.nih.gov/genbank/tsa) and were chosen for analysis if they met the following criteria: (1) The assembly program was listed; (2) the reads were Illumina paired-end reads; and (3) the published assembly contained at least 5000 contigs. TransRate assembly scores for this set of published assemblies ranged from 0.001 to 0.52 (Fig. 6A, light gray line). Each assembly was also subject to automated assembly score optimization producing optimized assembly scores that ranged from 0.001 to 0.6 (Fig. 6A, black line). Although some assembly scores showed little or no change following removal of low scoring transcripts, most improved when contigs below the learned cut-off were discarded (Fig. 6B).

It has been suggested that the transcriptomes from certain groups of organisms may be more difficult to assemble than others (Martin and Wang 2011). To investigate whether TransRate assembly scores varied for different taxa, the results were analyzed according to their major phylogenetic groups (Fig. 6C). For clades with more than 10 representative assemblies, no association between assembly quality and taxonomic group was found (Fig. 6C).

To determine whether any assembler consistently produced higher TransRate assembly scores on end user data sets, the performance of methods that had at least 10 assemblies was compared (Fig. 6D). In this test, Trinity, Oases, and SOAPdenovo-Trans all produced assemblies that spanned similar score ranges, with the highest mean score exhibited by Trinity (Fig. 6D). In contrast, Newbler, Agalma, and Trans-ABySS assemblies produced lower TransRate scores (Fig. 6D). However, caution should be exercised when interpreting these results as the user-modifiable settings and post-assembly processing steps were not reported for these published assemblies. Thus, the extent to which the TransRate assembly scores were influenced by changes in user-modifiable assembly parameters or post-assembly processing is unknown.

Because neither assembly method nor taxonomic group produced a major effect on the TransRate score of an assembly, we sought to determine whether the quality of the input read data was responsible for some of the variation in TransRate assembly scores. The read data for each assembly was analyzed using FastQC, and the resulting read-level metrics compared to the TransRate assembly scores of the assemblies generated using those reads. This revealed that neither the read length nor the percentage GC of the read data set exhibited any correlation with TransRate assembly score (Fig. 6E,F). However, significant associations were observed for both read quality (*r*^2^ = 0.27) (Fig. 6G) and the level of read-duplication in the data set (*r*^2^ = 0.1) (Fig. 6H). In Illumina sequencing, low read qualities are predominantly caused by errors in the sequencing process; common sources include over-clustering of the flow cell and phasing. In contrast, increases in read-duplication are caused by errors in the sample preparation stage. It occurs during the PCR amplification stage of the read library preparation, and is generally caused by either conducting the library preparation from too little starting material or by having a large variance in the fragment size such that smaller fragments become overrepresented during the limited cycle PCR. Although there is little correlation between the number of sequenced reads and the TransRate score of the assembled transcriptome (Fig. 6I), there is a clear association between the relative coverage implied by those reads and the TransRate score (*r*^2^ = 0.16) (Fig. 6J). In summary, the quality of the sequence reads, the number of reads per gene, and the quality of the input cDNA library (in order of relative contribution) explain 43% of the variance in de novo assembly quality. Thus, the quality of the input data is more important in determining the quality of a de novo assembly than the choice of assembly method that is used.

## Discussion

Here, we present TransRate a novel method for reference-free assessment and filtering of de novo assembled transcriptomes. Our method is focused on a clear definition of an optimal de novo assembled transcriptome, that it should be a complete and accurate representation of the transcripts encompassed in the raw read data. TransRate avoids conflating assessment of de novo assembly quality with other criteria (such as coverage of expected reference transcript subsets) that are not equivalent to correct or complete assembly of the input reads. Moreover, the method is not biased by expression level of the transcripts, and each transcript is weighted equally in the overall transcriptome assessment (unless the alternative abundance weighted metric is used). As the majority of published de novo assembled transcriptomes use Illumina paired-end sequencing, our analysis of the efficacy of TransRate is focused on this data type. However, the method is suitable for the analysis of other types of sequencing and thus is not restricted to use in the analysis of Illumina data.

TransRate is specifically designed to provide detailed insight into the quality of any de novo assembled transcriptome and each of its constituent contigs such that comparative analysis between assembly methods and post-assembly filtering of good and bad contigs can be performed. As TransRate does not use reference data sets in the evaluation of assemblies it is equally suitable for the assessment of assemblies of all types of RNA, including long noncoding RNA, mRNA, ribosomal RNA, and mixed RNA samples. Moreover, given multiple assemblies generated using the same input reads, TransRate can also be used to determine the assembly that best represents the input read data. Thus, TransRate could be used to help improve the performance of multiple different de novo transcriptome assembly algorithms. TransRate can also be used to filter out low scoring contigs; however, caution should be exercised here as application of filtering may result in removal of transcripts that have some utility. For example, transcripts with very low coverage are more likely to have low contig scores because of fragmentation and encapsulated bases in gapped regions; these transcripts, while incompletely assembled, may have utility in pathway reconstruction, quantitative expression analysis, or phylogenetic analysis. Similarly, transcripts with low *s*(*C*_seg_) scores are likely to represent chimeric transcripts. Here, although the transcript itself may be incorrectly assembled, the component segments of the transcript may themselves be correctly assembled and of utility if separated. To help users identify and diagnose likely assembly errors affecting low scoring contigs, TransRate provides each of the separate contig scores (in addition to the overall contig score). This information can be used to help resolve assembly errors on a contig-by-contig basis. Further investigation by systematically exploring a large range of read mapping parameters across a large range of read mapping algorithms and assembly tools may yield new ways to improve the performance of TransRate. This may improve the *s*(*C*_ord_) and *s*(*C*_cov_) measures that are affected by read coherency (Myers 2005), which may, in turn, suggest how the assemblies could be improved.

To help end users to interpret the TransRate scores that they obtain for their own assemblies and place them in context of previously published assemblies, we provide a meta-analysis of 155 published de novo assemblies. Here, a user generated de novo assembly with a TransRate score of 0.22 (optimized score of 0.35) would be better than 50% of published de novo assembled transcriptomes that have been deposited in the NCBI TSA. Through detailed analysis of these 155 published assemblies, we reveal that the quality of the input read data is the major factor determining the quality of any de novo transcriptome assembly, explaining more of the variance in quality between assemblies than the assembly method that is used.

## Methods

### Algorithm overview

TransRate is a reference-free qualitative assessment tool for the analysis of de novo transcriptome assemblies. TransRate requires one or more transcriptome assemblies and the reads used to generate those assemblies. TransRate aligns the reads to the assembly, processes those read alignments, and calculates contig scores using the full set of processed read alignments. TransRate classifies contigs into those that are well assembled and those that are poorly assembled, by learning a score cut-off from the data that maximizes the overall assembly score.

### Read alignment

Reads are aligned to a given assembly using SNAP v1.0.0 (Zaharia et al. 2011). Alignments are reported up to a maximum edit distance of 30. Up to 10 multiple alignments are reported per read where available (-omax 10), up to a maximum edit distance of 5 from the best-scoring alignment (-om 5). Exploration within an edit distance of 5 from each alignment is allowed for the calculation of MAPQ scores (-D 5). BAM-format alignments produced by SNAP are processed by Salmon (Patro et al. 2015). Separate strategies are used for abundance estimation and posterior read assignment. For abundance estimation, each mapped read is fractionally assigned to each potential contig of origin using Salmon (Patro et al. 2015) in a process that is analogous to the proportional assignment of the EM procedure used in RSEM (Li et al. 2010). For contig score evaluation, a different approach was taken in which a single assignment was produced for each read. Here, each read was assigned entirely to a single contig, but the probability of assignment for multimapping reads was sampled from the distribution of relative transcript abundances. Thus, during contig evaluation, each read is given an all-or-nothing assignment, with assignments sampled in proportion to the estimated abundances.

### Simulation of chimeric transcripts

The complete set of transcripts (*n* = 5917) for the *Saccharomyces cerevisiae* genome were downloaded from http://www.yeastgenome.org/. The transcripts were quantified and mRNA abundances recorded using Salmon, and the same set of reads used in the de novo assembly evaluation is described in “Analysis of assemblies generated from real reads.” To simulate transcript chimeras, 1000 transcripts were selected at random without replacement from the complete set of transcripts. Pairs of transcripts (*n* = 500) were fused in silico by concatenation of two of the randomly selected full-length transcript sequences head-to-tail. These 500 transcript chimeras were placed back into the reference transcriptome file (replacing both of their constituent transcripts) such that the transcriptome submitted to TransRate contained the 500 chimeric transcripts and the 4917 transcripts that were not chimeric (*n* = 5417). The transcriptome was subject to assessment with TransRate using the same set of RNA-seq reads. This process was repeated 20 times to obtain the results for the analysis of 10,000 chimeras. The *s*(*C*_seg_) score for each transcript chimera was compared to the difference in the relative abundance of the constituent transcripts in the chimera.

### TransRate contig scores

TransRate outputs scores for every contig. Here, an assembly consists of a set of contigs *C* derived from a set of reads, $\hat{R}$. Reads are aligned and assigned to contigs such that *R*_*i*_ is the set of reads assigned to *C*_*i*_. We propose that a correctly assembled contig derived from a de novo transcriptome assembly will have the following four intuitive properties:

The identity of the nucleotides in the contig will accurately represent the nucleotides of the true transcript *s*(*C*_nuc_). This score measures the extent to which the nucleotides in the mapped reads are the same as those in the assembled contig. If the mapped reads do not support the nucleotides of the contig, then this is likely because either the nonsupportive reads should map to a different contig or to a contig that is not represented in the assembly (a similar gene family variant, alternative allele, or other similarly encoded gene), or the assembled sequence is incorrect. In the case of the former, a missing contig (i.e., one that is not assembled) will negatively affect the score of the contig to which its reads incorrectly map. Although the contig to which they map may be correctly assembled, the negative score for this contig can be justified because the incorrectly mapped reads will render the abundance estimate of the assembled contig invalid. In the case of the latter, disagreement between the reads and the contig must be due to misassembly. To ensure that stochastic read errors that result in disagreement between a read and a contig do not affect the overall score for that contig, support for an alternative nucleotide sequence needs to be provided by multiple reads (see below).
The number of nucleotides in the contig will accurately represent the number in the true transcript, *s*(*C*_cov_). This score measures the proportion of nucleotides in the contig that have zero coverage, and thus have no supporting read data. If there are nucleotides in the contig that are not covered by any reads (regardless of the agreement between the reads and the sequence of the contig), then this should negatively affect the contig score.
The order of the nucleotides in the contig will accurately represent the order in the true transcript, *s*(*C*_ord_). This score measures the extent to which the order of the bases in contig are correct by analyzing the pairing information in the mapped reads. Here, if the orientation of the mapped reads does not conform to an expected mapping estimated from an analysis of a subsample of mapped read pairs, then these incorrectly mapping reads will negatively affect the contig score. Similarly, if the contig could have been extended, i.e., there are read pairs that map such that one read is present near a terminus of the contig and its pair is not mapped and would be expected to map beyond the scope of the contig, then such cases indicate that the contig does not use all of the available reads, and thus is incompletely assembled. This metric is informative for the identification of partially assembled transcripts.
The contig will represent a single transcript, *s*(*C*_seg_). This score measures the probability that the coverage depth of the transcript is univariate, i.e., that it represents an assembly of a single transcript and not a hybrid/chimeric assembly of multiple transcripts expressed at different expression levels. Here, the per-nucleotide coverage depth of the contig must be best modeled by a single Dirichlet distribution (described below). If the contig is better modeled by the product of two or more Dirichlet distributions, then this indicates that two or more contigs with different transcript abundances have been erroneously assembled together.

The TransRate contig score is the product of the scores for each of these properties using the aligned reads as evidence. These four properties are evaluated next.

### Calculation of *s*(*C*_nuc_)

The alignment edit distance is used to quantify the extent to which the contig sequence is correct. The alignment edit distance is the number of changes that must be made to the sequence of a read in order for it to perfectly match the contig sequence. Here, the edit distance of an aligned read $r_{ij}\in R_{i}$ is denoted as $e_{r_{ij}}$, and the set of reads that cover nucleotide k(k∈[1,n]) as ϱk. The maximum possible edit distance for an alignment is limited by the read alignment algorithm (described in “Read alignment”) and is denoted as $\hat{e}$. Support for the contig provided by the reads is then evaluated as $1-e_{r_{ij}}/\hat{e}$ for each $r_{i}\in \varrho k$, and the mean of all support values is used to calculate *s*(*C*_nuc_).

### Calculation of *s*(*C*_cov_)

This score is evaluated as the fraction of nucleotides in the contig that receive at least one mapped read irrespective of the agreement between the read and the contig.

### Calculation of *s*(*C*_ord_)

The pairing information of the mapped reads is used to evaluate this score. To determine the parameters of the read library preparation, a randomly selected subsample of 1% of all mapped read pairs are analyzed. From these alignments, the orientation of the paired-end reads is determined, and the mean and standard deviation of the fragment size is inferred. All read pair alignments are then classified according to whether they are plausible given the estimated parameters of the library preparation and assuming that the assembled contig is correct. A read pair is considered correct if the following criteria are met: (1) Both reads in the pair align to the same contig; (2) the relative orientation of the reads in the pair is consistent with the inferred library preparation parameters; and (3) the relative position of the reads is consistent with the mean and standard deviation of the inferred fragment size. *s*(*C*_ord_) is then evaluated as the proportion of all mapped read pairs that are correct.

### Calculation of *s*(*C*_seg_)

The per-nucleotide read coverage data is used to evaluate this score. To evaluate the probability that the contig originates from a single transcript (i.e., it is not chimeric), a Bayesian segmentation analysis of the per-nucleotide coverage depth is performed. For a correctly assembled contig, it is assumed that the distribution of per-nucleotide coverage values in that contig is best described by a single Dirichlet distribution, i.e., all nucleotides in the same transcript should have the same expression level, and thus should be best modeled as a stochastic sample from a single distribution. In contrast, a contig that is a chimera derived from concatenation of two or more transcripts will have per-nucleotide coverage values that are best described by two or more different Dirichlet distributions. The probability that the distribution of per-nucleotide read coverage values comes from a single Dirichlet distribution is evaluated using a Bayesian segmentation algorithm previously developed for analysis of changes in nucleotide composition (Liu and Lawrence 1999). To facilitate the use of this method, the per-nucleotide coverage along the contig is encoded as a sequence of symbols in an unordered alphabet by taking log_2_ of the read depth rounded to the nearest integer. As the probability will be a value between 0 and 1, this probability is used directly as *s*(*C*_seg_).

### TransRate assembly score

The aim of the TransRate assembly score is to provide insight into the accuracy and completeness of any given assembly. Thus, the assembly score weights equally a summary statistic of the TransRate contig scores and the proportion of the input reads that are contained within this assembly. We note here that alternative methods for summarizing contig scores that weight contig scores by their expression level would produce different results. However, such schemes would not be consistent with the problem definition and aim of TransRate: to assess the accuracy and completeness of a de novo assembled transcriptome using only the input reads. This score assumes that an ideal assembly will contain a set of contigs that represent unique and complete transcripts to which all of the reads used to assemble those transcripts can be mapped. The TransRate assembly score (*T*) is evaluated as the geometric mean of the mean contig score and the proportion of read pairs that map to the assembly such that

(1) $$T=\sqrt{{\left(\overset{n}{\underset{c=1}{\prod }}s(C)\right)}^{\frac{1}{n}}R_{valid}},$$

where

(2) $$s(C)=\;s(C_{\mathrm{nuc}})s(C_{\mathrm{cov}})s(C_{\mathrm{ord}})s(C_{\mathrm{seg}}).$$

### The abundance-weighted TransRate score

An abundance-weighted contig and assembly score are also provided by TransRate. The contig score is evaluated as

(3) $$s_{w}(C)={s(C)}^{1+\log_{n}(TPM+1)},$$

where *s*(*C*) is as defined in equation 2; and *TPM* is the transcripts per million transcripts value assigned to that contig by Salmon. Under this framework; highly abundant transcripts that have assembly errors are penalized more heavily than low abundance transcripts with the same errors. The abundance-weighted assembly score (*T*_*w*_) is thus evaluated as

(4) $$T_{w}=\sqrt{{\left(\overset{n}{\underset{c=1}{\prod }}s_{w}(C)\right)}^{\frac{1}{n}}R_{valid}}.$$

### Analysis of assemblies generated from real reads

To demonstrate the utility TransRate contig and assembly scores using real data, TransRate was applied to publicly available benchmark assemblies from two previous analyses (Davidson and Oshlack 2014; Xie et al. 2014). One set comprised different assemblies generated for rice (*Oryza sativa*) and mouse (*Mus musculus*) using the Oases, Trinity, and SOAPdenovo-Trans assemblers (Xie et al. 2014). The other set comprised assemblies for human (*Homo sapiens*) and yeast (*Saccharomyces cerevisiae*) that had been assembled with Oases and Trinity (Davidson and Oshlack 2014). These assemblies were chosen as they have previously been independently used in benchmark comparisons, and each of the species has a completed annotated reference genome available. In all cases, TransRate was run with the published reads and the published assembly as input.

### Independence of score components

Correlation between the contig score components was measured for the assemblies from real data. To prevent larger assemblies from biasing the results, 5000 contigs were sampled at random from each assembly. These contigs were used to calculate a Spearman's rank correlation coefficient using R version 3.1.1 (R Core Team 2014). The correlation between any two score components was taken as the mean of the correlation across all data sets.

### Identification of reconstructed reference transcripts

The full set of coding and noncoding transcripts for each species was downloaded from Ensembl Genomes version 25 (ftp://ftp.ensemblgenomes.org/pub/release-25/). Assembled contigs were then identified by BLAST searching the reference data set for the corresponding species using bidirectional blastn local alignment with an e-value cut-off of 10^−5^ (BLAST+ version 2.2.29) (Camacho et al. 2009). Only reciprocal best hits were retained for further analysis.

### Assembly from simulated read data

For each species, a total of 10 million mRNA molecules were simulated from the full set of annotated mRNAs from the Ensembl reference with exponentially distributed expression values using the flux simulator v1.2.1 (Griebel et al. 2012). mRNA molecules were uniform-randomly fragmented and then size-selected to a mean of 400 nt and standard deviation of 50 nt. From the resulting fragments, 4 million pairs of 100-bp reads were simulated using the default error profile included in flux-simulator. An assembly was generated from these simulated reads using SOAPdenovo-Trans with default parameters.

### Calculation of contig accuracy

Accuracy was calculated by comparing contigs assembled from simulated data to the set of transcripts from which the read data were simulated. Reciprocal best BLAST hits were identified, and the accuracy of each contig assembled from simulated read data was evaluated as the contig *F*-score where

(5) $$\text{Contig precision}=\frac{\text{Number of correct nucleotides in contig}}{\text{Number of nucleotides in contig}},$$

(6) $$\text{Contig recall}=\frac{\text{Number of correct nucleotides in contig}}{\text{Number of nucleotides in reference transcript}},$$

(7) $$\text{Contig}\;F\text{-score}=2\left(\frac{\left(\text{contig precision}\right)\left(\text{contig recall}\right)}{\left(\text{contig precision}+\text{contig recall}\right)}\right).$$

Spearman's rank correlation coefficient between the contig *F*-score and the TransRate contig score was calculated using R version 3.1.1 (R Core Team 2014). The same contigs were also subject to analysis using RSEM-eval, and the relationship between contig impact score and contig *F*-score was analyzed using the same method.

### Constructing a benchmark data set of TransRate scores

A survey of the range of assembly scores for published de novo transcriptome assemblies was conducted by analyzing a subset of transcriptome assemblies from the Transcriptome Shotgun Archive (http://www.ncbi.nlm.nih.gov/genbank/tsa). De novo assembled transcriptomes were used in this analysis only if paired-end reads were provided, the assembler and species were named in the metadata, and the assembly contained at least 5000 contigs (TransRate has no minimum or maximum contig requirements, but a minimum number of 5000 was imposed to ensure sufficient raw data was available for analysis). For each of these test data sets, the assembly and reads were downloaded. TransRate was run on all assemblies, and FastQC version 2.3 (http://www.bioinformatics.babraham.ac.uk/projects/fastqc) was used to evaluate the quality of the read data sets.

### Software availability

TransRate is written in Ruby and C++ and makes use of the BioRuby (Goto et al. 2010) and BAMtools (Barnett et al. 2011) libraries. The source code is available in a compressed archive in Supplemental File S1 and at http://github.com/Blahah/transrate and is released under the open source MIT license. Binary downloads and full documentation are available at http://hibberdlab.com/transrate. The software is operated via a command line interface and can be used on OSX and Linux. TransRate can also be used programmatically as a Ruby gem.
